# The Living and Working Together Perspective on Creativity in Organizations

**DOI:** 10.3389/fpsyg.2019.02733

**Published:** 2019-12-17

**Authors:** Diletta Gazzaroli, Caterina Gozzoli, Gonzalo Sánchez-Gardey

**Affiliations:** ^1^Department of Psychology, Università Cattolica del Sacro Cuore, Milan, Italy; ^2^Management Department, Universidad de Cádiz, Cádiz, Spain

**Keywords:** creativity, living and working together in organizations, identity, otherness, work purpose, organizational culture of difference

## Abstract

Although creativity represents a cornerstone for organizations that want to keep up with competitors, customers, and the current socio-economic context, there is a dearth in the literature of systemic and comprehensive models focused on the complexity and addressing several dimensions and factors. In this context, we propose the perspective of “working and living together in organizations” to enrich the scientific dialogue with a proposition that aims to hold together different variables of interaction and relationship between different parts of the organization (Gozzoli, 2016a,b). In fact, according to our previous studies (Frascaroli et al., 2016; Gorli et al., 2016; Marta et al., 2016; Saita et al., 2016; Tamanza et al., 2016), a generative living and working together environment is itself directly linked to creativity and innovative processes. This is because in a generative living and working together environment relationality – that is, the possibility of exchange among workers mediated by the object of work – is enabled. With this study, we intend to provide a contribution to the creativity study field, applying our perspective to an extensive level of analysis. The model was tested using the Exploratory Structural Equation Modeling methodology with EQS-6.3. Our results found some interesting elements in support of the theory behind this study.

## Introduction

For organizations, the ability to innovate is crucial to long-term performance (Martínez-Tur et al., 2001; Tomic, 2010; Osman, 2013). Thus, creativity and innovation processes allow organizations to be more prepared to deal with market’s unpredictability and customers’ requests.

According to Tomic (2010), innovation can be defined as the successful implementation of creative ideas (Woodman et al., 1993; Wood, 2003; Gaspersz, 2005) as a consequence of the desire to grow, keep up with competitors, and adapt to changing customer needs, as well as the result of people satisfying their curiosity by finding new concepts or optimizing existing ones (Porter, 1990; Hopp, 1998; Amabile and Conti, 1999). Organizational creativity can be interpreted as the creation of a valuable, useful new product, service, idea, procedure, or process by individuals working together in a complex system (Woodman et al., 1993). In this sense, creativity is seen as “the cornerstone of innovation” (Tomic, 2010, p. 322).

Creative professionals should thus be able to contribute substantially to the pursuit and achievement of high-quality standards through the generation of ideas (Chai et al., 2005). In fact, team creativity, and thus organizational creativity itself, can be understood as “the joint novelty and usefulness of ideas regarding products, processes, and services” (Hoever et al., 2012, p. 983).

According to the literature (Oldham and Cummings, 1996), creativity is a multidimensional construct, resulting from the presence and combination of multiple skills: (1) fluidity, understood as the ability to produce a large number of ideas; (2) flexibility, the ability to imagine ideas that are different from each other; (3) processing, the ability to develop and enhance new ideas; (4) originality, the ability to generate ideas that are unique; and (5) functionality, defined as the ability to generate ideas of value that are appropriate and useful.

Because of the multidimensionality of creativity, several authors (Deci and Ryan, 1987; Mumford, 2000; Zhou, 2003; Gaspersz, 2005; Rice, 2006) have underlined the need to consider different factors in a creative work process, including:

1. The inclusion of all human resources in the process of innovation and knowledge sharing.
2. The promotion of divergent thinking in a work environment that is “risk-taking” and free of constrictions.
3. Free exchange among professionals with different educational and training backgrounds.
4. Open communication oriented toward the construction of flows that allow for the exchange of information and feedback.
5. Promotion of a climate of tolerance of failure.
6. A clear and shared vision of objectives.
7. Being able to perceive a link between role and personal/professional resources.
8. Perceiving social recognition within the professional context, as the relational dimension of interaction is a key component in daily professional life.
9. Perceiving high levels of security with respect to the maintenance of the work and with a professional growth.

However, according to literature (see Tomic, 2010), only few models combine knowledge derived from different subfields. We present these briefly below.

In the social psychology field, we can find Amabile’s componential theory of creativity (Amabile, 1983), which states that creative processes imply three components: domain-relevant skills (developed through education and work experience); creativity-relevant skills (such as cognitive thinking style and personality characteristics); and intrinsic task motivation (such as the attitude toward a task), which interacts with the external social environment in which an individual operates. Amabile et al. (2005) theorized an “organizational affect-creativity cycle,” which assumes that positive and negative affects lead to cognitive variation, which can increase or decrease creativity.

Ramamoorth et al. (2005) suggests that the perceived obligation to innovate is influenced by three organizational factors: psychological contract, job design, and organizational justice.

A model that can be classified within both social and organizational psychology is Borghini’s (2005). According to this circular model, cultural integration can enhance creativity. In fact, when cultural integration supports a sharing process between employees, it promotes creativity. Conversely, when cultural integration encourages the codification of knowledge, which may result in organizational rigidities, it may limit creativity.

DiLiello and Houghton (2006) developed a model that can be placed within the context of personality, social, and organizational psychology. It suggests that individual creativity can be influenced by two kinds of factors: a strong self-leadership that increases one’s sense of one’s own innovative and creative potential, and the perception of strong support in practicing innovation and creativity.

Woodman and Schoenfeldt (1989) suggest that creative actions are the results of a person’s behavior in a given situation (that either facilitates or inhibits creativity). Later, Woodman et al. (1993) proposed that group creative behavior depends on individual creative behavior, group composition, group characteristics, group processes, and contextual influences. In turn, group creative behavior is input for organizational creative behavior, and together with contextual influences it may lead to a creative outcome.

According to Tomic (2010), in all these models, there is a need for more systemic and comprehensive approaches, combining the main constructs involved at different organizational levels: individual, group and organizational. Finally, Tomic (2010) highlights that little is known about the validity and predictive value of the models in the literature.

Moreover, even if analyzing the most recent works, an increase in studies on individual and collective creativity can be traced; there is still a lack of studies on organizational creativity and empirical works that consider complex variables together (Jeong and Shin, 2017).

In this sense, Gozzoli’s (2016a,b) perspective on “Living and working together in organizations” (from now on LWTO) could be significant to enrich the scientific dialogue on organizational creativity. This perspective, as emerges from various empirical qualitative studies (Gozzoli and Frascaroli, 2012; Gozzoli et al., 2014, 2018a,b; Scaratti et al., 2014, 2017; Frascaroli et al., 2016; Gorli et al., 2016; Marta et al., 2016; Saita et al., 2016; Tamanza et al., 2016; D’Angelo et al., 2018), aims to consider variables of interaction and relationship involved in the definition of the living and working together style to better understand their influence on creativity and innovative processes. In fact, a generative living and working together environment, where professionals are allowed to deal with the Otherness with an open dialogue, is itself directly linked to creativity and innovation processes.

As stated by Gozzoli, LWTO has a twofold nature, involving both constraints and obligations on the one hand (“in organizations you do not choose your colleagues; you are forced to work with them”) and resources and opportunities on the other (“you can get along and reinforce each other”). It is precisely the way in which the relationship with Otherness is developed that can provide opportunities to grow, to innovate, to activate creative processes, and to manage complex challenges. Provided that in organizations, people live together to produce services or goods, LWTO may therefore be the type of relation and interaction among identity, otherness, work purpose, and organizational culture of diversity. “It is the relational dimension between the other and me (each of us with our own system of meanings, values, powers, practices, and cultures) mediated by the work purpose (meant at the same time as possible identification object and driving force for an action and relation with the other), within a specific organizational culture of diversity, which tells me about various living and working together styles^1^” (Gozzoli, 2016b, p. 225). Identity symbolizes all that is known and familiar that which defines and provides “safety.” Otherness, with its own system of meanings, values, powers, practices and cultures of reference, represents what is not known, is scary and calls the familiar into question^2^. The work purpose in this triangulation becomes an element of identification and motivation to act versus non-identification. Thus, work purpose is the third element (concrete and symbolic at the same time), where identity and otherness must confront each other in an organization. In this “space of comparison” between identity and otherness, difference has at its disposal a field of action, where the conflict may or may not be acted upon without being pervasive or destructive^3^. This triangulation, with regard to an organizational context, must be situated and understood within a specific organizational culture that informs how that matter should be treated. For organizational life, this means that if the “subjects” involved are in an organizational culture of difference that allows for the possibility of an encounter with difference, the object of work can be redefined through dialogue and the conflict can be generative. Otherwise, if the “subjects” involved are in an organizational culture of difference in which there is no space for the recognition of difference and dialogue related to it, the object of work is unlikely to be recognized and shared, and the conflict is likely to be destructive. The organizational culture of difference (and its more or less explicit and conscious management) provides the context that accelerates or inhibits the ability to treat that difference and thus influence the well-being of people and the capacity for innovation.

In other words, identity, otherness, work purpose, and organizational culture of diversity “turn out to be precious to understand more or less generative forms to live and work together in groups within organizations and have comprehension elements to be able to intervene and activate transformative processes. Their intersection outlines them” (Gozzoli, 2016b, p. 226).

### The Proposition

**Figure 1:**
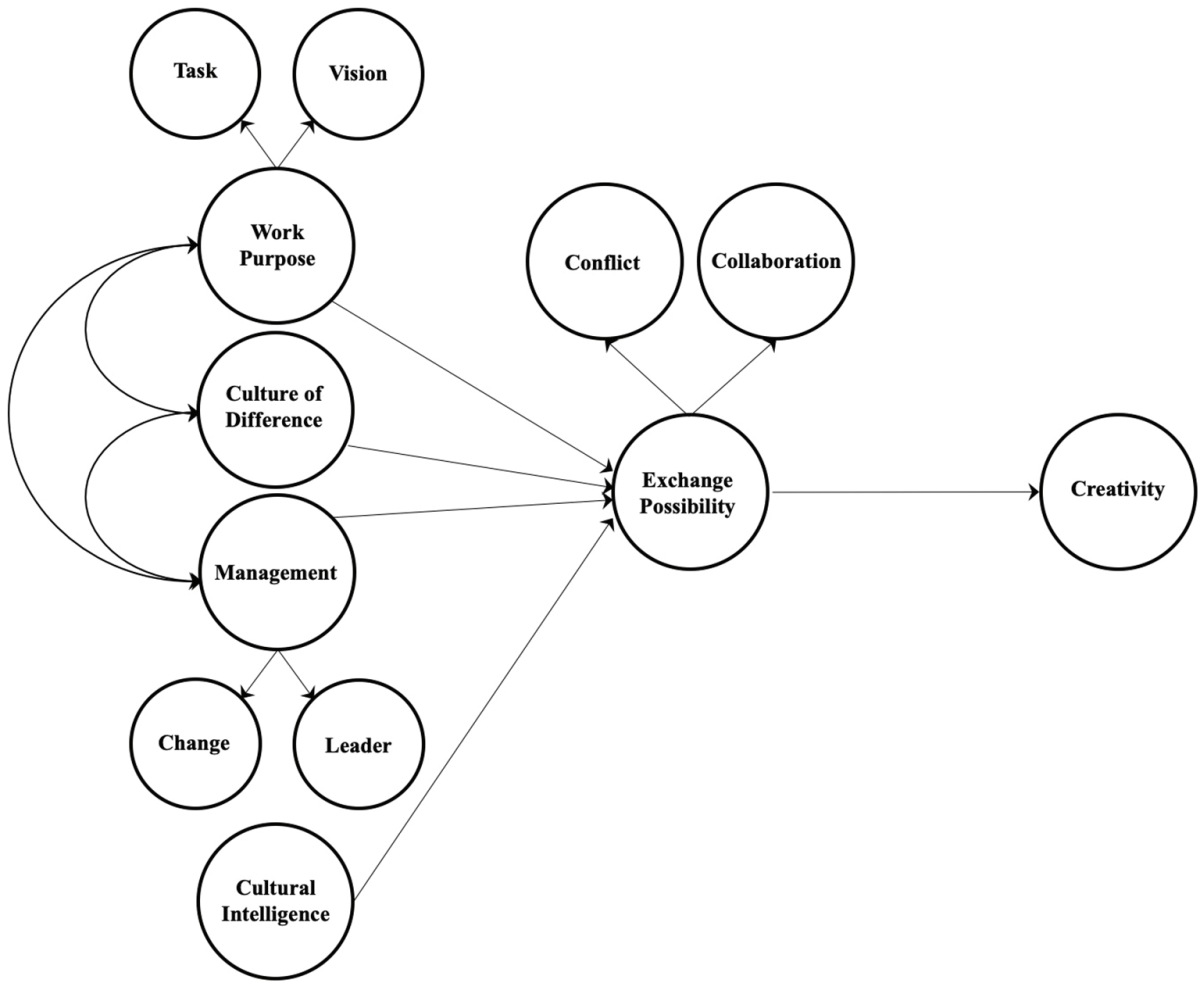
Living and working together model to explain creativity.

The LWTO perspective is inherently connected with organizational creativity and innovative processes. With this study, we intend to provide a contribution to the creativity study field, applying this perspective to an extensive level of analysis. Below (Figure 1), we will explain the ones that we considered appropriate to choose for a first proposal because it allowed us to reach a more complex vision and combine together different constructs and different organizational levels (individual, group, and organizational).

Creativity here is explained by the exchange possibility, which is an indirect measure of the quality of the relationship between identity and otherness. Thus, according to the LWTO approach, the presence of generative conflict and areas of collaboration between professionals represents the ability to access the integration of perspectives, knowledge, and different skills, and consequently to promote creative processes. Among the exogenous variables of the model, we find four constructs predicting exchange possibility: cultural intelligence, organizational culture of difference, work purpose, and organizational management. Cultural intelligence is a variable chosen to operationalize the quality of the relationship between identity and otherness because it refers to professionals’ individual capability to get in touch with the difference and thus – according to our theoretical approach – to feel safer when getting in touch with otherness. In the model, the other three exogenous constructs are connected to each other. Organizational culture of difference is the factor that organizationally informs how to handle the relationship with Otherness and so – as stated by our approach – to feel allowed (or not allowed) to get in touch with the difference to build new, shared visions. To operationalize the organizational culture of difference, organizational management was chosen because it represents an indicator of how an organization translates into actions its assumptions about how to handle diversity. Finally, we have work purpose, which – according with our approach – represents the third element where identity and otherness must confront each other. This means that when the work purpose – its sense, its sustainability and its consistency in terms of professional expectations – is shared, it is possible to maintain the relation even when conflicting.

In summary, in light of the model, the aim of this work is to deepen how this framework provides a reliable theoretical foundation for understanding creativity and innovation in organizations.

## Materials and Methods

### Aims and Scope

In accordance with the literature presented and our theoretical perspective, this study – assuming the viewpoint of individual’s representation – aims to deepen how the *living and working together style* – defined by the quality of the relationship between identity and otherness, clear and shared work purpose and the organizational culture of difference – is linked with organizational creativity and innovation.

### Sample and Procedure

Data were collected by filling out the questionnaire in a paper version (and, if required by the organization, in the presence of a researcher) or in an online version on the Qualtrics platform (which could be accessed through a dedicated link).

The questionnaire was composed of closed questions and generally required a time of between 35 and 40 min to complete.

A total of 835 questionnaires were collected and were entered in a first database for preliminary analysis. Some were discarded because they did not meet the basic criteria (60% of missing answers on the whole questionnaire).

There were 816 participants in the study. Among those who answered the question, 33.8% are male and 66.3% are female. The clear majority of participants who responded (792) are Italian (90.7%). The age of participants who responded to this question (786) is between 20 and 63 years, and the subjects are distributed in a sufficiently balanced manner among the various age groups: 25.4% in the 20–32 age range; 27.6% in the 33–41 age range; 22.0% in the 42–48 age range; and 24.9% in the 49–63 age range. The average age is 40.5 years (SD = 10.24).

As reported, among those who responded (776), distribution between the types of organization showed that the percentage of companies was slightly lower (27.7%) compared to cooperatives (36.7%) and assisted living facilities (35.6%). With regard to the professional role, among those who responded (789), the largest group was in the category of care assistants/healthcare professionals (33.1%). These are followed by roles having decision making and planning functions in the group (12.7%), those with a socio-humanistic education (13.1%) and those with managerial functions (11.2%).

The different organizations that participated in the study were predominantly contacted in the Lombardy region (85%).

The sampling criterion was one of convenience (no particular inclusion criteria were adopted except for the companies that had to be involved in production). We get in touch with the organizations thanks to the network of two federations of professionals.

### Sample and Procedure

#### Cultural Intelligence

It is an indicator of the individual capability to understand, act and manage effectively in culturally diverse settings, which is one of the preconditions for Identity to be open to connect with Otherness (Ang et al., 2007; Gozzoli and Gazzaroli, 2018). This construct is measured with the Cultural Intelligence Scale (Ang et al., 2007; Gozzoli and Gazzaroli, 2018).

#### Exchange Possibility Between Identity and Otherness

It could be seen as a factor that indirectly informs on the quality of the relationship between Identity and Otherness. The quality of this relationship is a crucial construct in our theoretical frameworks, because it implies the possibility to avoid disruptive conflict and promote generative and non-judgmental debate processes. In fact, the quality of relationships resulting from the culture of difference and triangulation between “identity-otherness-work” indicates how much space there is for the possibility of a destructive/generative conflict and collaboration in order to access the possibility of innovating and protecting the well-being of the people within the organization. Exchange possibility cannot be measured directly. More precisely, it is the factor resulting from two first-order factors: collaboration and conflict. That is the reason why we choose “Collaboration” and “Social Conflict” subscale of the Rahim Organizational Conflict Inventory – I (Rahim, 1995), as edited in the Italian version by Majer (1995).

#### Work Purpose

Work purpose, as an element of triangulation between identity and otherness, must be clear and shared. Thus, according to our theoretical framework, work purpose helps to keep the focus on the working process because it is the third element where identity and otherness must confront each other. This construct is measured with two Team Climate Inventory subscales (Ragazzoni et al., 2002): “vision” and “task.” The development of the Team Climate Inventory (TCI) is based on the four-factor model of West (1990) according to which the innovative capacity of a group is linked to the constructs of “vision,” “participative safety,” “task orientation” and “support for innovation.” In fact, according to this model, if the group has a clear and shared vision of the goals, its efforts will be directed and facilitated in the development of effective work processes. Similarly, group members should feel free to offer their contribution and should be supported in the efforts required to achieve set standards. For the present study, the “vision” and “task orientation” subscales of the short Italian version already used and validated by Ragazzoni et al. (2002) seemed particularly suited. The decision to select these two subscales is linked to the conceptualization and the role attributed to the work in our theory of reference. The work purpose, in fact, must be clear, shared and the focus in orienting actions.

#### Organizational Culture of Difference

Organizational culture of difference (OCD) is an indicator of the assumptions on diversity that a group has not only been able to give itself but has also validated and translated into strategies, recognizing in them an intrinsic value. According to our theoretical framework, it is the internalization of this system of meanings that makes it possible to understand how to behave in the organization. This construct is measured with the Diversity Perspective Questionnaire (Podsiadlowski et al., 2012).

#### Organizational Management

Organizational management can be considered an indicator of the processes related to how an organization translates into actions its assumptions about how to handle diversity. Organizational management is measured with two Organizational Check-up System subscales: “change” and “leadership” (Leiter and Maslach, 2000; Borgogni et al., 2005).

#### Creativity

Creativity as organizational outcome, allows the organization to evolve and maintain a competitive position in the market. It is characterized by a processual dimension and a temporal prefiguration on the medium/long term. This construct is measured with a scale formulated “*ad hoc*” on the basis of the definition of creativity of Oldham and Cummings (1996). We used a scale formulated *ad hoc* because we did not find any scale satisfactory with our perspective and criteria on creativity; for example, the Creative Product Semantic Scale (O’Quin and Besemer, 1989) for the analysis of creative products, the Employee Creativity Scale (Tierney et al., 1999) and the Creative Potential and Practiced Creativity Scale (DiLiello and Houghton, 2006) that are focused on individual creativity.

### Measures

Some guidelines (Fowler, 2002) were followed in the construction of the questionnaire: we used validated scales whose reliability has been shown in previous studies; we avoided the use of open-ended questions; and we used a Likert scale with at least five steps to maximize the variance; we chose the scales closest to our constructs.

It is also important to highlight that all the measures proposed in the questionnaire are self-report measures based on individual perceptions even when they concern group or organizational dimensions. The following scales were used to analyze the model.

#### Cultural Intelligence

The scale consists of 20 items, referring to the four factors underlying the construct of cultural intelligence, outlined below. “Metacognitive CQ”: skills related to the thought processes by which people acquire and understand cultural knowledge, 4 items; “Cognitive CQ”: body of knowledge of the rules, practices and conventions of different cultures gained through education and personal experience, 6 items; “Motivational CQ”: the ability to direct one’s attention and one’s resources to learning and operating in a situation characterized by cultural difference, 5 items; “Behavioral CQ”: the ability to exhibit appropriate verbal and non-verbal behavior during interactions with people of different cultures, 5 items. Each subscale is composed of items that measure the construct in a direct way (the highest degree of agreement corresponds to the maximum degree of consensus with the detected perspective). All items are closed questions on a five-point Likert scale.

#### Exchange Possibility Between Identity and Otherness

“Collaboration” and “Social Conflict” subscale of the Rahim Organizational Conflict Inventory – I (1995), as edited in the Italian version by Majer (1995) are structured as follow: “Collaboration,” 6 items; “Social Conflict,” 6 items. Each subscale is composed of items that measure the construct in a direct way (the highest degree of agreement corresponds to the maximum degree of consensus with the detected perspective). All items are closed answers on a five-point Likert scale.

#### Work Purpose

According to the proposal of Ragazzoni et al. (2002) “vision” and “task orientation” subscales of the short Italian version of the Team Climate Inventory are structured as follows: “Vision”: the degree of clarity with which the goals and vision of the group are believed to be defined, shared, attainable and measurable (10 items); and “Task Orientation”: commitment by the group to achieve the highest possible levels of performance with the support of procedures for monitoring the process (8 items). Each subscale is composed of items that measure the construct in a direct way (the highest degree of agreement corresponds to the maximum degree of consensus with the detected perspective). All items are closed answers on a five-point Likert scale.

#### Organizational Culture of Difference

Diversity Perspective Questionnaire (Podsiadlowski et al., 2012), hereinafter DPQ, is a self-reporting instrument that measures the perception of organizational culture of difference and its management. Compared to the original version of the instrument proposed by Podsiadlowski et al. (2012), the present study considered only the five organizational vignettes. The vignettes provide for the choice of one of the scenarios described in them, referring to five different perspectives with which an organization can relate to inherent difference, ranging from the maximum rejection of difference to its appreciation. The five perspectives are characterized as indicated below:

1. “Reinforcing Homogeneity Perspective”: refers to avoiding or even rejecting a diverse workforce.
2. “Color Blind Perspective”: focuses on equal employment opportunities, without acknowledging potential differences.
3. “Fairness Perspective”: ensures equal and fair treatment by addressing the need for specific support for minority groups.
4. “Access Perspective”: sees diversity as a business strategy that provides access to a diverse customer base and international markets.
5. “Integration and Learning Perspective”: suggests that everyone can benefit from a diverse work environment.

The five vignettes are introduced by the following statement: “In the following, you will find five short descriptions of organizational scenarios. Choose the one that, in your opinion, best represents your organization.”

#### Organizational Management

Organizational Management is measured with two Organizational Check-up System subscales: “Change” and “Leadership” (Leiter and Maslach, 2000; Borgogni et al., 2005). The original version of the instrument consists of 68 items, organized into an “MBI-General Survey” and an “Areas of Worklife Survey.” For the present study, the subscales “change” and “leadership” from the Areas of Worklife Survey seemed particularly suited, as they can provide information on the translation mode in terms of practices and actions of the organizational culture’s assumptions. In the original proposal, the subscales are structured as follows. “Change”: measures the perception of change over the past 6 months, 10 items; “Leadership”: refers to the judgment of supervisors and the quality of communication with the organization’s top management, 5 items. The scales are composed of items that measure the construct in a direct way (the highest degree of agreement corresponds to the maximum degree of malaise) and from items that measure the construct through an “inverse” formulation (the maximum degree of agreement corresponds to the minimum degree of malaise). All items are closed answers on a five-point Likert scale.

#### Creativity

This construct is measured with a scale formulated “*ad hoc*” on the basis of the definition of creativity of Oldham and Cummings (1996). We used a scale formulated *ad hoc* because we did not find any scale coherent with our perspective on creativity. According to Oldham and Cummings (1996), creativity is a multidimensional construct, the result of the presence and the combination of multiple skills: fluidity, understood as the ability to produce a large number of ideas; flexibility, the ability to imagine ideas that are different from each other; processing, the ability to develop and enhance new ideas; originality, the ability to generate ideas that are unique; Functionality, the ability to generate ideas of value that are appropriate and useful. The formulation of the scale provides for people to answer while thinking about their organizations with respect to the five components identified by the authors. Each subscale is composed of items that measure the construct in a direct way (the highest degree of agreement corresponds to the maximum degree of consensus with the detected perspective). All items are closed answers on a five-point Likert scale.

### Data Analysis

The proposed model was tested using the Exploratory Structural Equation Modeling (ESEM) methodology with EQS-6.3 (Byrne, 2006; Marsh et al., 2014). ESEM is an overarching integration of the best aspects of CFA/SEM and traditional EFA, providing confirmatory tests of a-priori factor structures, relations between latent factors and multigroup/multioccasion tests of full (mean structure) measurement invariance.

A two-stage procedure was used to test the hypotheses using the Bentler-Weeks approach to SEM (Bentler, 1995). The first phase involved testing the measurement properties of the constructs; we conducted a confirmatory factor analysis (CFA) for all scales. The aim of this first step is to check the degree of adequacy of the scales with respect to the sample participating in the study. We then tested structural relationships among constructs to verify the proposed relationships.

To verify the degree of fit, the following criteria were taken into consideration: goodness-of-fit statistics [Chi-square statistic (*χ*^2^), Non-Normed Fit Index (NNFI), Comparative Fit Index (CFI), Standardized Root Mean Square Residual (SRMR), Root Mean Square Error of Approximation (RMSEA), Raykov’s Reliability RHO (*ρ*)], discriminant reliability, and factor loadings.

The results obtained for these two tests are explained in the following sections.

## Results

### Measurement Properties Testing

#### Cultural Intelligence

To check the degree of adequacy of the scales with respect to the sample participating in the study, we conducted a Confirmatory Factor Analysis (Table 1).

**Table 1:** Fit indices – four-factor CQS model.

*N*	*χ*^2^ (gdl)	NNFI	CFI	SRMR	RMSEA	*ρ*
755	765.399^a^ (164)	0.92	0.93	0.05	0.07^b^	0.95

a *The probability value for the chi-square statistic is 0.00000. However, *χ*^2^ is sensitive to the sample size; with a large sample size it is highly probable to have a *p* < 0.05, even if the model fits the data (Corbetta, 1992)*.

b *MacCallum et al. (1996) suggest that values from 0.80 to 0.10 indicate a mediocre, but still acceptable fit*.

In light of the results and items’ saturation (all between 0.628 and 0.880), and as proposed by Ang et al. (2007), the final version is composed of 20 items and 4 different theoretical dimensions (Metacognitive, Cognitive, Motivational, Behavioral) that correlate with each other.

#### Exchange Possibility Between Identity and Otherness

For this study, the factors “collaboration” and “social conflict” were considered for the definition of the construct “exchange possibility.” To check the degree of adequacy of the scales with respect to the sample participating in the study, we conducted a Confirmatory Factor Analysis (Table 2).

**Table 2:** Fit indices – “collaboration” and “social conflict” subscale.

Subscale	***χ***^2^ (gdl)	NNFI	CFI	SRMR	RMSEA	*ρ*
**Collaboration**
All items	346.816^b^ (13)	0.77	0.86	0.08^c^	0.18	0.86
**Removing items with lower saturation^c^**						
Without item 8	250.394^b^(8)	0.78	0.88	0.07	0.19	0.86
Without item 8-13	32.904^a^ (4)	0.95	0.98	0.02	0.09^d^	0.85
**Social conflict**
All items	242.779^b^(13)	0.77	0.90	0.07^c^	0.15	0.93
**Removing items with lower saturation^c^**						
Without item 5	125.506^b^(8)	0.84	0.91	0.05	0.14	0.80
Without item 5-19	13.202^b^(4)	0.98	0.99	0.02	0.05	0.80
Without item 5-19-9	3.754^b^(1)	0.98	0.99	0.01	0.06	0.80

a *The probability value for the chi-square statistic is 0.00000. However, x2 is sensitive to the sample size; with a large sample size it is highly probable to have a p-value <.05, even if the model fits the data (Corbetta, 1992)*.

b *MacCallum et al. (1996) suggest that values from 0.08 to 0.10 indicate a mediocre, but still acceptable fit*.

c *The numbering of the items shown here corresponds to the order in which they are presented in the original scale*.

d *MacCallum et al. (1996) suggest that values from 0.08 to 0.10 indicate a mediocre, but still acceptable fit*.

For the “Collaboration” subscale, in light of the final attempt results and items’ saturation (all between 0.564 and 0.835), the decision was made to include in the final version of the scales the following items: collaboration1, collaboration2, collaboration14, collaboration18, and collaboration20. For the “Social Conflict” subscale, in light of the final attempt results and items’ saturation (all between 0.538 and 0.890), the decision was made to include in the final version of the scales the following items: social-conflict4, social-conflict10, social-conflict12, and social-conflict16.

Exchange possibility between identity and otherness, as a latent construct, results from these first-order factors: collaboration and conflict. To check the degree of adequacy of the scales with respect to the sample participating in the study, we conducted a Confirmatory Factor Analysis (Table 3).

**Table 3:** Fit indices – “relationship.”

Attempt	*χ*^2^ (gdl)	NNFI	CFI	SRMR	RMSEA	*p*
All items	184.072^a^(39)	0.93	0.95	0.05	0.07	0.86

a *MacCallum et al. (1996) suggest that values from 0.08 to 0.10 indicate a mediocre, but still acceptable fit*.

In light of the results and items’ saturation (all between 0.530 and 0.879), in the final version all items were considered.

#### Work Purpose

Work purpose is measured with two Team Climate Inventory subscales (Ragazzoni et al., 2002): “Vision” and “Task.” To check the degree of adequacy of the scales with respect to the sample participating in the study, we conducted a Confirmatory Factor Analysis (Table 4).

**Table 4:** Fit indices – “vision” and “task orientation” subscales.

Subscale	*χ*^2^ (gdl)	NNFI	CFI	SRMR	RMSEA	*p*
**Vision**
All items	854.302^a^(34)	0.76	0.82	0.08^b^	0.18	0.90
**Removing items with lower saturation^b^**						
Without item 9	572.720^a^(26)	0.81	0.86	0.07^b^	0.16	0.90
Without item 9-8	470.103^a^(16)	0.82	0.88	0.06^b^	0.17	0.90
Without item 9-8-10	311.999^a^(13)	0.85	0.90	0.06^b^	0.17	0.90
Without item 9-8-10-7	277.824^a^(8)	0.82	0.90	0.06^b^	0.21	0.90
Without item 9-8-10-7-5	23.197^a^(4)	0.98	0.99	0.02	0.08^c^	0.90
**Task orientation**
All items	165.779^*^ (19)	0.95	0.96	0.03	0.09^b^	0.92

a *MacCallum et al. (1996) suggest that values from 0.08 to 0.10 indicate a mediocre, but still acceptable fit*.

b *The numbering of the items shown here corresponds to the order in which they are presented in the original scale*.

c *MacCallum et al. (1996) suggest that values from 0.08 to 0.10 indicate a mediocre, but still acceptable fit*.

For the “Vision” subscale, in light of the final attempt results and items’ saturation (all between 0.639 and 0.906), the decision was made to include in the final version of the scales the following items: vision1, vision2, vision3, vision4, and vision6. For the “Task” subscale, in light of the results and items’ saturation (all between 0.643 and 0.872), the decision was made to include in the final version of the scales the following items: task11, task12, task13, task14, task15, task16, task17, and task18.

The work purpose, as a latent construct, results from these first-order factors: vision and task orientation. To check the degree of adequacy of the scales with respect to the sample participating in the study, we conducted a Confirmatory Factor Analysis (Table 5).

**Table 5:** Fit indices – “work purpose.”

Attempt	*χ*^2^ (gdl)	NNFI	CFI	SRMR	RMSEA	*p*
all items	440.258^a^(60)	0.93	0.94	0.05	0.09^b^	0.94

a *MacCallum et al. (1996) suggest that values from 0.08 to 0.10 indicate a mediocre, but still acceptable fit*.

b *The numbering of the items shown here corresponds to the order in which they are presented in the original scale*.

In light of the results and items’ saturation (all between 0.641 and 0.895), in the final version all the items were considered.

#### Organizational Management

Organizational Management is measured with two Organizational Check-up System subscales: “Change” and “Leadership” (Leiter and Maslach, 2000; Borgogni et al., 2005). To check the degree of adequacy of the scales with respect to the sample participating in the study, we conducted a Confirmatory Factor Analysis (Table 6).

**Table 6:** Fit indices – “change” and “leadership” subscales.

Subscale	*χ*^2^ (gdl)	NNFI	CFI	SRMR	RMSEA	*p*
**Change**
All items	311.933^a^(34)	0.93	0.94	0.04	0.10^b^	0.93
**Removing items with lower saturation^b^**						
Without item 2	230.853^a^(26)	0.94	0.96	0.03	0.10^b^	0.93
Without item 2–3	161.684^a^(19)	0.95	0.97	0.03	0.09^b^	0.93
**Leadership**
All items	175.993^a^(8)	0.90	0.95	0.04	0.16	0.92

a *MacCallum et al. (1996) suggest that values from 0.08 to 0.10 indicate a mediocre, but still acceptable fit*.

b *The numbering of the items shown here corresponds to the order in which they are presented in the original scale*.

For the “Change” subscale, in light of the final attempt results and items’ saturation (all between 0.694 and 0.820), the decision was made to include in the final version of the scales the following items: change1, change4, change5, change6, change7, change8, change9, and change10. For the “Leadership” subscale, in light of the results and items’ saturation (all between 0.689 and 0.860), the decision was made to include in the final version of the scales the following items: leadership1, leadership2, leadership3, leadership4, and leadership5.

Organizational management, as a latent construct, results from the first-order factors of change and leadership. To check the degree of adequacy of the scales with respect to the sample participating in the study, we conducted a Confirmatory Factor Analysis (Table 7).

**Table 7:** Fit indices – “management processes.”

Attempt	*χ*^2^ (gdl)	NNFI	CFI	SRMR	RMSEA	*ρ*
All items	469.966^a^(72)	0.93	0.95	0.04	0.09^b^	0.95

a *MacCallum et al. (1996) suggest that values from 0.08 to 0.10 indicate a mediocre, but still acceptable fit*.

b *The numbering of the items shown here corresponds to the order in which they are presented in the original scale*.

In light of the results and items’ saturation (all between 0.694 and 0.872), in the final version all items were considered.

#### Creativity

To check the degree of adequacy of the scales with respect to the sample participating in the study, we conducted a Confirmatory Factor Analysis (Table 8).

**Table 8:** Fit indices – “creativity” subscale.

Attempt	*χ*^2^ (gdl)	NNFI	CFI	SRMR	RMSEA	*ρ*
all items	65.548^a^(4)	0.97	0.99	0.01	0.10^b^	0.96

a *MacCallum et al. (1996) suggest that values from 0.08 to 0.10 indicate a mediocre, but still acceptable fit*.

b *The numbering of the items shown here corresponds to the order in which they are presented in the original scale*.

In light of the results and items’ saturation (all between 0.856 and 0.950), the decision was made to include all items in the final version.

#### Model Testing

According to the results (Table 9), the model does not show particularly good fit indices. After analyzing *β* parameter, the decision was made to create a second model deleting the associations between:

1. “OCD and DM” factor and exchange possibility (*β* = 0.01);
2. CQS’ factors and exchange possibility (*β* = 0.01–0.07).

**Table 9:** Fit indices model – creativity outcome.

*χ*^2^ (gdl)	NNFI	CFI	SRMR	RMSEA	*ρ*
4848.761^a^(1797)	0.88	0.89	0.13	0.05	0.96

a *MacCallum et al. (1996) suggest that values from 0.08 to 0.10 indicate a mediocre, but still acceptable fit*.

**Figure 2:**
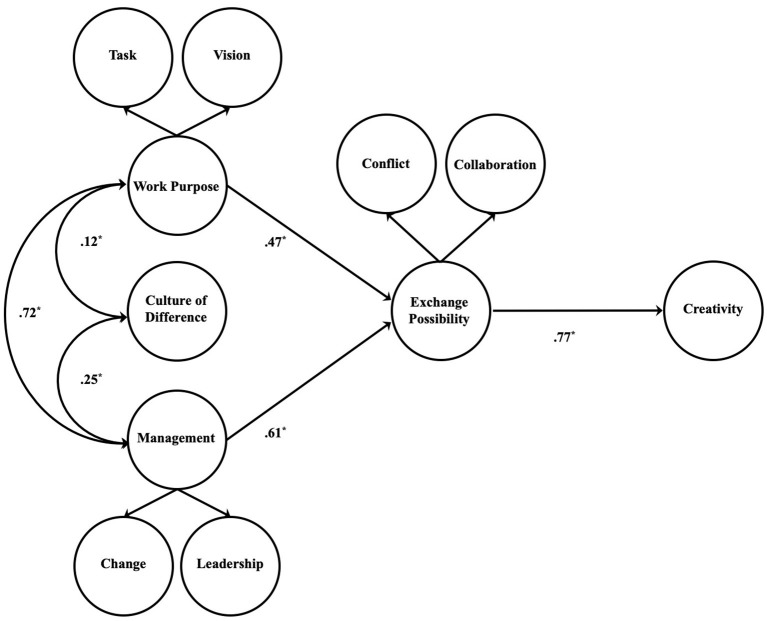
Living and working together final model.

According to the results (Table 10, Figure 2), has good fit indices, which are statistically significant and satisfy *β* parameter.

**Table 10:** Fit indices model modified –creativity outcome.

*χ*^2^ (gdl)	NNFI	CFI	SRMR	RMSEA	*ρ*
2416.854^a^(796)	0.91	0.92	0.08^b^	0.06	0.97

a *MacCallum et al. (1996) suggest that values from 0.08 to 0.10 indicate a mediocre, but still acceptable fit*.

b *The numbering of the items shown here corresponds to the order in which they are presented in the original scale*.

These results show that the theoretical model could be considered an interpretative hypothesis that discreetly fits the data. All of the model’s associations, regardless of their strength, are significant for *p* < 0.001.

When considering the relations that constitute this model, it is possible to draw some inferences. The first of these is about the correlations among the antecedent exogenous variables. Results confirm the relations proposed, indicating that the three exogenous variables correlate with each other in the same direction, as shown by the positive sign. Particularly strong is the correlation between organizational management and work purpose, with a *β* value of 0.72. Correlations between OCD and DM with organizational management and work purpose, although present, are weaker (*β* = 0.12; *β* = 0.25). Regarding the association between organizational management and work purpose with the exchange possibility, the model seems to be confirmed. This study, in particular, highlights the strength of this association, especially with regard to organizational management (*β* = 0.61). Finally, in regard to the outcome variable, as shown by the *β* value of 0.77, the impact of the exchange possibility on creativity is quite high.

## Discussion

Based on the analysis of the materials, the final model suggests that the Working and Living Together in Organizations perspective can contribute to the issue of organizational creativity.

As stated in the introduction of the paper, in organizations the relationship between Identity and Otherness is triangulated with a third concrete and symbolic element: work purpose. Work purpose in this triangulation becomes an element of identification and motivation to act. This triangulation, with regard to an organizational context, must be situated and understood within a specific organizational culture that informs on how that matter should be treated. Organizational management, in this sense, could be considered a source of information on the translation in terms of practices and actions of the assumptions of the organizational culture. Therefore, the organizational culture of difference and the more or less explicit and conscious practices for its translation into actions constitute the context that can accelerate or inhibit the possibility to deal with the difference. This, in turn, could have an impact on organizational creativity.

As evidenced by the final version of the models in this study, it was necessary to delete the association between the OCD factor and the exchange possibility, because the *β* parameter could not be considered satisfying. The fact that this association has been deleted, does not necessarily means the absence of an impact of the OCD factor.

In support of this hypothesis, there are correlations present (albeit not particularly high) with the organizational management and the work purpose. Thus, these correlations, indicating the presence of a link between the constructs, confirm results already found in the literature (Pil and Cohen, 2006; Avery et al., 2007; Chavez and Weisinger, 2008; Kravitz, 2008; McKay et al., 2008; Talke et al., 2011; Reiter-Palmon and Murugavel, 2018) that *in terms of creativity and innovation, the organizational culture of difference may play a role and needs to be deepened*. Thus, OCD is the result of the internalization of systems of meanings, which allows professionals to orient themselves within the organization. At a conscious and an unconscious level, the organizational culture defines the boundaries of what can be considered legitimate. In other words, the organizational culture of difference “crosses” the organization, and the correlation (in this sense) could be seen as a “proof” of its relevance. Another reason that might be connected with the lack of an association between the organizational culture of difference and the exchange possibility is the *novelty of the topic*, especially in the Italian context.

Examining the model in depth, it can be noted that, compared with the work purpose, the organizational management has a higher impact on the exchange possibility. This finding could be explained by the fact that *management processes are those that more explicitly relate, not only to the management of individual professional, but also to the overall system of human resources*. Very often, the figure of the leader plays a key role in promoting a vision of difference as a resource, by fostering relationships with Otherness, spurring on a reflective attitude and thus promoting a creative environment. Even the perception of change represents an immediate source of information for professionals. Through it, professionals can get feedback on resource allocation and the direction followed by the organization in terms of new possibilities and interests.

With regard to the association between exchange possibility and creativity, results seem to confirm the assumptions of our perspective. Based on the analysis, we can say that *in the way the relation with the Otherness is lived, in terms of cooperation and non-destructive conflict, lies the possibility of growth, innovation, creative processes and thus managing complex challenges.*

A last important reflection is need with regard to cultural intelligence. According to the results, cultural intelligence does not seem relevant in terms of impact in our model. This result may depend on the nature of the construct. In fact, Cultural Intelligence refers to the individual’s capability to function and manage effectively in culturally diverse settings, particularly ethnic ones. This result may indicate that, *in Italian organizational contexts, there is still little space to recognize diversity and perceive it as a* “*matter of fact.*” This limited and “fragmented” vision toward difference could explain the reason why there are still not enough reflections and awareness on the meanings and relevance ascribed to the relationship with Otherness when talking about creativity and innovation processes.

## Conclusion

As pointed out earlier, organizational creativity can be understood as “the joint novelty and usefulness of ideas regarding products, processes, and services” (Hoever et al., 2012, p. 983).

As a multiple construct (Oldham and Cummings, 1996), it is the result of the presence and combination of multiple skills and factors (Woodman et al., 1993).

Creativity, as an organizational outcome, allows the organization to evolve and maintain a competitive position in the market.

However, according to the literature (Tomic, 2010; Jeong and Shin, 2017), only a few models combine knowledge derived from different subfields and little is known about the validity and predictive value of these models. Moreover, even if analyzing the most recent works, an increase in studies on individual and collective creativity can be traced there is still a lack of studies on organizational creativity and empirical works that consider complex variables together.

For all these reasons, with this study – assuming the viewpoint of individuals’ representation – we decided to deepen the contribution to the creativity and innovation study field of Gozzoli’s “LWTO” perspective. This perspective, indeed, shows how the *living and working together style* – defined by the quality of the relationship between Identity and Otherness, clear and shared Work Purpose and the Organizational Culture of Difference – is linked with organizational creativity and innovation.

As shown in the discussion, creativity actually seems to be influenced by the exchange possibility between Identity and Otherness, which is predicted by four interconnected exogenous variables: cultural intelligence, organizational culture of difference, work purpose and organizational management. In other words, this study confirms at an extensive level Gozzoli’s perspective, which states that the quality of the relationship between Identity and Otherness, clear and shared Work Purpose and the Organizational Culture of Difference are linked with organizational creativity and innovation. Thus, the work purpose in this triangulation makes it possible to find a space to “get in touch” and a possible agreement. With regard to organizational management, its impact can be explained only by the fact that creativity is characterized by a procedural dimension where a key role is played by lines of action and strategic choices defined by the management. Talking about organizational culture of difference, although the association between the organizational culture of difference factor and exchange possibility was deleted from the model, this does not necessarily mean that organizational culture of difference is an irrelevant variable. The presence of correlations with the other exogenous variables of the model is a first indicator of its relevance. Correlations, showing a link between these constructs, are a first demonstration that the organizational culture of difference “crosses,” influences the organization. Even more important is the consideration on the novelty of this construct in Italy.

We can say that the way of living in relation with Otherness, in terms of cooperation and non-destructive conflict, represents the possibility of growth, innovation, creative processes and thus managing complex challenges.

As shown by the study’s results, it is important to understand the process by which an organization, through the organizational culture, defines the meanings that connote work purpose, management processes and exchange possibilities, because all these factors together may determine the degree of creativity in organizations.

Finally we would like to emphasize that in previous qualitative studies (Gozzoli et al., 2014, 2018a; Scaratti et al., 2014, 2017; Frascaroli et al., 2016; Gorli et al., 2016; Marta et al., 2016; Saita et al., 2016; Tamanza et al., 2016) we found that the living and working together style that characterizes an organization determines the organizational generative possibility in creative and relational terms. Having found that only a non-destructive living and working together style can protect this generative dynamic, we believe that it is important to continue our studies to better understand what happens in destructive living and working together styles.

In conclusion, we can say that LWTO represents a perspective that allows for a better understanding of the variables that influence organizational creativity and offers a key to possible interventions aimed at modifying/consolidating the dynamics that characterize the living and working together style and, consequently, the possible degree of creativity.

### Limitations and Future Directions

Firstly, although the sample size in quantitative terms can be considered appropriate, it is not sufficient to ensure adequate and heterogeneous representativeness of the national working population. In fact, most of the organizations involved in our studies have a very homogeneous Human Resources composition (especially regarding nationality). It would be important to investigate how the model works with a different sample composition. Moreover, a sample with a more heterogeneous Human Resources population may offer new and different data. In this regard, promoting an in-depth analysis of professionals’ representations of the concept of difference could be helpful to understand when professional perceive this capability as useful to enhance creativity.

This study investigates the impact of the organizational culture of difference and diversity management on factors that characterize organizational life through professionals’ individual representations. It could be interesting to compare data with agreement scores at the group and organizational level. Given the complexity and sensitivity of the issue and the potential impact of social desirability or psychological resistance in providing the answers, it may be useful to continue the research considering qualitative deepening to grasp the phenomenon’s different shades.

Future studies might involve a comparison between different kind of organizations. In light of the recent evolution of the socio-economic context, it would be interesting to understand whether there are differences. Effectively, in service realities the work is generally characterized by a strong symbolic charge, whereas in production enterprises it is generally characterized by a strong business charge. However, although service realities and production enterprises are characterized by “products” with a different nature, they are both still called to promote creativity processes to respond to competitive standards in order to “stay in the market.”

## Ethics Statement

This study was carried out in accordance with the recommendations of “Commissione Etica per la Ricerca in Psicologia, Università Cattolica del Sacro Cuore’ with written informed consent from all subjects. All subjects gave written informed consent in accordance with the Italian Legislative Decree 30 June 2003 n. 196. The written informed consent is reported below: “I agree to the proposal to participate in the research study. My agreement is an expression of a free decision, not influenced by promises of economic benefits or otherwise, nor from obligations to the principal investigator of the study. I am aware of being free to withdraw from the study at any time I want. Moreover, I am aware that I’m not supposed to give any reasons for my decision to withdraw from the study. I was given the opportunity to ask questions about the aims and methods of the study and my rights as a participant in the research. I understood all the information and explanations that I have been given and I had enough time to consider my participation in this study. According to the Italian Legislative Decree 30 June 2003 n. 196, I agree that my personal data will be used for specific research purposes within the limits and in the manner please explain in the information document.” The protocol was approved by the “Commissione Etica per la Ricerca in Psicologia.”

## Author Contributions

DG worked as a researcher. CG contributed to scientific supervision. GS-G worked as an analysis consultant.

### Conflict of Interest

The authors declare that the research was conducted in the absence of any commercial or financial relationships that could be construed as a potential conflict of interest.

## Footnotes

^1^For a detailed description of these styles, see Gozzoli (2016b).

^2^For us Otherness is all that involves difference from the self; therefore, it can be the individual, a group or a collective vision.

^3^In our perspective (which is part of a constructionist approach) the work purpose can be understood as a kind of boundary object (Star, 2010).
